# The Belfast Youth Development Study (BYDS): A prospective cohort study of the initiation, persistence and desistance of substance use from adolescence to adulthood in Northern Ireland

**DOI:** 10.1371/journal.pone.0195192

**Published:** 2018-05-23

**Authors:** Kathryn Higgins, Aisling McLaughlin, Oliver Perra, Claire McCartan, Mark McCann, Andrew Percy, Julie-Ann Jordan

**Affiliations:** 1 Centre for Evidence & Social Innovation, School of Social Sciences, Education & Social Work, Queen’s University Belfast, Belfast, United Kingdom; 2 School of Nursing & Midwifery, Queen’s University Belfast, Medical Biology Centre, Belfast, United Kingdom; 3 MRC/CSO Social and Public Health Sciences Unit, University of Glasgow, Glasgow, United Kingdom; The Pennsylvania State University, UNITED STATES

## Abstract

**Background:**

Substance misuse persists as a major public health issue worldwide with significant costs for society. The development of interventions requires methodologically sound studies to explore substance misuse causes and consequences. This Cohort description paper outlines the design of the Belfast Youth Development (BYDS), one of the largest cohort studies of its kind in the UK. The study was established to address the need for a long-term prospective cohort study to investigate the initiation, persistence and desistance of substance use, alongside life course processes in adolescence and adulthood. The paper provides an overview of BYDS as a longitudinal data source for investigating substance misuse and outlines the study measures, sample retention and characteristics. We also outline how the BYDS data have been used to date and highlight areas ripe for future work by interested researchers.

**Methods:**

The study began in 2000/1 when participants (n = 3,834) were pupils in their first year of post-primary education (age 10/11 years, school year 8) from over 40 schools in Northern Ireland. Children were followed during the school years: Year 9 (in 2002; aged 12; n = 4,343), Year 10 (in 2003; aged 13; n = 4,522), Year 11 (in 2004; aged 14; n = 3,965) and Year 12 (in 2005; aged 15; n = 3,830) and on two more occasions: 2006/07 (aged 16/17; n = 2,335) and 2010/11 (aged 20/21; n = 2,087). Data were collected on substance use, family, schools, neighbourhoods, offending behaviour and mental health. The most novel aspect of the study was the collection of detailed social network data via friendship nominations allowing the investigation of the spread of substance use via friendship networks. In 2004 (school year 11; respondents aged 14), a sub-sample of participants’ parents (n = 1,097) and siblings (n = 211) also completed measures on substance use and family dynamics.

**Results:**

The most recent wave (in 2010/2011; respondents aged 20/21 years) indicated lifetime use of alcohol, tobacco and cannabis among the cohort was 94, 70 and 45 per cent, respectively. The paper charts the development of drug use behaviour and some of the key results to date are presented. We have also identified a number of key areas ripe for analysis by interested researchers including sexual health and education.

**Conclusions:**

We have established a cohort with detailed data from adolescence to young adulthood, supplemented with parent and sibling reports and peer network data. The dataset, allowing for investigation of trajectories of adolescent substance use, associated factors and subsequent long-term outcomes, constitutes an important resource for longitudinal substance misuse research. A planned further wave as the cohort enter their late twenties and potential to link to administrative data sources, will further enrich the datasets.

## Introduction

Substance misuse remains an intractable public health issue with significant costs for individuals, their families and wider society. In the US, the abuse of alcohol, tobacco and illicit drugs costs more than $700 billion annually [1–3]. In the UK, drug and alcohol abuse has been estimated to cost £36 billion a year [4]. Substance use is generally initiated during the adolescent years. In the US, by the 12^th^ grade (age 18), about half of adolescents have abused an illicit drug at least once [5]. On average, 21% of the boys and 15% of the girls aged 15–16 years old in Europe have tried illicit drugs at least once in their lifetime [6]. For the majority of youth, experimentation with illegal substances during adolescence is transient. For others, it can lead to a wide range of health and social problems which can continue into adulthood including offending behaviour [7], academic under achievement [8], mental health problems [9] and substance abuse [10].

The Belfast Youth Development Study (BYDS) was established to address the need for a longitudinal prospective cohort study to investigate the initiation, persistence and desistance of substance use behaviours (particularly problem drug use) from early adolescence to adulthood, with a view to informing interventions to prevent or reduce the harms associated with substance misuse. The study was created to comprehensively map out adolescent drug use behaviours (from 11 years of age onwards) and how they may change over time and to investigate the psychological and socialisation agents (including the family, peers, schools and neighbourhoods) that shape these drug use pathways. While focusing primarily on substance use, a wealth of information was collected on other aspects of young people’s lives including free time activities, music listened to, delinquency, income and relationships, with the purpose of investigating processes that could explain or moderate initiation, persistence and desistance of drug use. The study also aimed to assess variations and determinants of these other outcomes (such as mental health, academic and vocational outcomes) for young people. Therefore, the study has a wider scope than drug use and can be described as a study of adolescent development.

The first phase of BYDS included five annual waves of data collection of cohort members (Table 1)–a period encompassing the five years of compulsory post-primary school (school years 8–12) for participants (early-mid adolescence; 10–15 years of age). Data collection focused on key social processes which play a pivotal role during adolescence. The family is one of the earliest sources of socialisation, and various structural elements and processes can influence adolescent substance use including parent-child attachment, parental monitoring and household composition [11, 12]. Many studies rely on child reports of family dynamics, however the BYDS also included a family survey of parents and older siblings in 2004 to include a two generation design (parent-child dyads), providing data which can address issues such as discrepancies in parent-child reports [13]. Schools also play a pivotal role in adolescent development and factors such as behaviour and attitudes towards school, educational aspirations and school attachment/connectedness have been associated with long-term outcomes [14]. The BYDS sample included 42 schools in total thus providing sufficient variability to examine the relative influence of both school and individual level variables on outcomes.

**Table 1:** Number of participants in each wave.

	Study Wave						
	Phase 1 School Years					Phase 2 Youth Transitions and Emerging Adulthood	
	1	2	3	4	5	6	7
Year of data collection	2000/2001	2002	2003	2004	2005	2006/2007	2010/2011
Respondent age	10/11 years	12 years	13 years	14 years	15 years	16/17 years	20/21 years
Academic school year	Year 8	Year 9	Year 10	Year 11	Year 12		
Eligible participants	4,410	5,216	5,239	5,254	5,155	4,189	4,180
Respondents	3,834 (86.9%)	4,343 (83.3%)	4,522 (86.3%)	3,965 (75.5%)	3,830 (74.3%)	2,335 (55.7%)	2,087 (49.9%)
Refusal	239 (5.4%)	308 (5.9%)	322 (6.1%)	254 (4.8%)	304 (5.9%)	373 (8.9%)	420 (10%)
Absent	337 (7.6%)	565 (10.8%)	395 (7.5%)	1,035 (19.7%)	1,021 (19.8%)	na	na
No answer*	na	na	na	na	na	na	457 (10.9%)
Wrong contact detailsǂ	na	na	na	na	na	na	176 (4.2%)
No completion after contact+	na	na	na	na	na	1,481 (35.4%)	1,040 (24.9%)
Left/moved school	na	89	215	304	463	na	na
Deceased	0	0	0	0	1	2	9

Note. Percentages are based on eligible participants. Eligible participants were all students enrolled in their schools participating year group including all recently enrolled students (even if they had not participated in previous waves). Students who had left a school were removed from the list of eligible participants. The eligible participant list also includes those who were unable to take part due to industrial action. Eligible participants total includes respondents, refusal, absent, no answer, wrong contact details, and no completion after contact. The source of these numbers was the student registry at each school. Total number of respondents = 5,809.

* = Respondents were invited to take part in wave 7 of the study by telephone (they were accessed via school/college in years 1–6);

^ǂ^ = Respondents were accessed via school in waves 1–5 and further education colleges in wave 6; in wave 6 they were asked to provide contact details (home address, phone number, email) to enable follow-up in wave 7; as the 7^th^ wave of data collection was 4–5 years after contact details were provided, some of these details were no longer correct (i.e. 176 respondents);

^+^ = Respondents in wave 7 were contacted via letter, email or phone call; respondents in wave 6 were contacted via higher education colleges and provided with a survey to return (via post) to the research team.

A unique aspect of BYDS Phase 1 (first five waves i.e. the compulsory school years; respondents aged 11–15 years), was the collection of social network data within each school; this allows the influence of peer networks on the initiation and spread of substance use behaviours among friendship networks to be measured. The impact of the peer group on youth as they transition from early to mid/late adolescence is well documented [15], however, few other studies to date have collected detailed peer network data to the same extent as BYDS. The quality of network data held by BYDS is also high (i.e. less than 20% missing data) compared to other surveys that collected peer network data such as the National Longitudinal Study of Adolescent Health [16]. In the BYDS, each participant was asked to nominate their best friend in the school year, and up to nine other friends–thus giving information on friendship networks within each school. As nominated friends were also participating in the study, it is possible to use the data collected to gather information concerning characteristics and behaviours of individuals in a friendship network over time using first-person information (i.e. information provided directly by the nominated friend). This is an innovative feature of the study insofar many other studies that have investigated peer influence in adolescence have often relied on the respondent’s report of his/her peers’ behaviour and characteristics [17,18] a report that may be subject to bias [19]. Therefore, BYDS provides a valuable resource to investigate the spread of behaviour through peer networks.

As the BYDS sample transitioned from compulsory schooling to further education and employment (Phase 2- waves 6 & 7; respondents aged 16–21 years) (Table 1), the aims of the study also transitioned to focus on key factors which were pertinent in their lives, in line with the extant literature. Data collection from 16–21 years focused on the early adult sequelae of adolescent substance use behaviour; the interactions between substance use patterns and mental health; the extent to which the transition from school to employment, training or unemployment affects substance use; pro-social and anti-social behaviour; and the formation of romantic experience and substance use.

Knowledge generated from the study has capacity to inform the international, national and local evidence bases in this area given the robustness of its design. A unique aspect of the BYDS longitudinal data is that it enables researchers to track youth outcomes over seven time points within a ten year period. As a result, the data are suitable for the application of sophisticated statistical models such as growth curve modelling. For example, the BYDS data have been used to examine different classes of offending trajectory and predictors of those trajectories [20]. The need for longitudinal studies of young people was particularly acute in the specific historic context in which BYDS began: Northern Ireland had entered a post-conflict transition following the Belfast Agreement. The background to the study was therefore a period of expected fast changes to society in Northern Ireland. In particular, different authors and studies had suggested that Northern Ireland would face a range of social problems that had been ignored or suppressed by the conflict and its dynamics: among these was adolescent drug use [21]. The examination of risk factors in a geographical area where rapid political changes were taking place provided an added bonus in terms of the unique opportunity to investigate the initiation, persistence and desistance of substance use among youth in a population undergoing transitions.

The aim of this paper is to describe the purpose and design of the BYDS cohort study. We also present a thematic review of how the BYDS data have been used to date and identify topics ripe for future analyses.

## Materials and methods

### Ethics

At its initiation in 2000, BYDS predated the Office for Research Ethics Committees Northern Ireland (ORECNI) (HPSS RECs were fully operational from May 2004). As a result the study was subject to university ethical review. Ethical issues were clearly identified, considered and protocols established to protect respondents through standard project management and governance procedures. The BYDS phase 2 (2006–2011), was reviewed and approved by the then School of Sociology, Social Policy and Social Work, Queen’s University Belfast ethics committee.

### Sample recruitment and attrition

In BYDS, a recruitment strategy was adopted to include a combination of both urban and rural areas. All secondary schools in Belfast and two intermediate townlands (Ballymena and Downpatrick) with rural catchment areas were invited to participate in the study. In wave 1 (school year 8; respondents aged 10–11 years old), thirty-nine (71%) out of an invited 55 schools participated. Two thirds of the schools in Belfast agreed to participate (7 refused), all of the schools in Downpatrick took part and three quarters of schools in Ballymena (3 refused). Reasons for schools non-participation varied from school to school and included timetable pressures, lack of interest and a wish for the school not to be associated with the drug and alcohol elements of the survey. In each school, all children in the first school year group were included in the study. Letters were sent to parents explaining the study and they were given the option to ‘opt out’ or remove their child from the study. Children were invited to complete a paper copy of the questionnaire in school and were given the option to refuse to participate. Children who had difficulties completing the survey were assisted by researchers. There were 4,411 pupils in the participating schools, 3,834 (87%) of whom completed a questionnaire at baseline/wave 1 (school year 8; respondents aged 11 years old).

In wave 2 (school year 9; respondents aged 12 years old) of the study, a further four secondary schools in Belfast as well as a number of non-mainstream education facilities (e.g. Education Other than School Projects, Educational and Behavioural Difficulty Units) joined the study. This was to obtain information on young people who may be excluded from mainstream education or were receiving further support, particularly those pupils from BYDS participating schools who transferred to alternative education provision. Young people attending these institutions are referred to in subsequent publications as the ‘High-Risk Booster sample’.

Cohort members entered and left the study as they moved to or from participating schools (Table 1). Three schools participated in industrial action in wave 4 and did not participate in the study (school year 11; 14 years old); two of these returned the next year (wave 5) and one withdrew. This led to a modest reduction in completion rates. The school year-based sampling strategy, combined with the addition and temporary withdrawal of some schools led to a complex pattern of entry and attrition compared to that obtained from a study which uses baseline enrolment and follows up individuals. For the first six waves (respondents aged 10–17 years), the sampling frame was all pupils in the corresponding year group at participating schools, regardless of whether or not any individual or school did or did not participate previously. Information on home address of the participants was only collected in waves 4, 5, 6 and 7 (respondents aged 14–21 years old), to enable follow up outside of school; hence some loss to follow up occurred where earlier entrants left participating schools. For the 7th wave (respondents aged 20/21 years old), only respondents who had provided contact details in previous waves were invited to participate.

In addition to the main cohort follow up, a family study was conducted in the summer of 2004 (between school years 4 and 5). This survey collected information on substance use and family dynamics from both parents of a cohort member (whenever possible) as well as an older sibling [22]. Parents of the BYDS cohort were contacted by post to request consent for their address information for the purposes of the Family Survey. This yielded a self-selecting sample of 938 parental addresses (20% of all eligible families in the 2004 BYDS cohort), a total of 1,097 parental interviews were completed with 721 individual households. Interviewees were asked to nominate the main care giver within the household, with two-thirds of the sample nominating themselves as the main care giver and 370 interviewees considered themselves to the ‘other’ care giver. The main carer interviews are approximately 15% of the total cohort size. Non-response at household level was due to refusal (8%), movers (8%) and non-contact (7%). Five households were comprised of siblings-only and they were excluded from the sample. Family members of 721 children in the cohort were interviewed. In 345 households, one parent was interviewed and 59 siblings; and in 376 households, both parents were interviewed and 148 siblings. The total number of completed interviews was 1,309. Information was collected on parents’ and siblings’ alcohol and drug use, parenting styles and family dynamics. These data were collected between waves 4 (school year 11; respondents aged 14 years old) and 5 (school year 12; respondents aged 15 years old) of the main study. To date, there has been no longitudinal follow up of the family study participants. (See [22] for more information).

### Measures

The BYDS cohort members responded to a range of measures throughout the seven waves of the BYDS providing data on their substance use (lifetime use, substance related problems), families (parental monitoring, attachment, household composition), schools (academic activities, attachment to school) and neighbourhoods (social control, disorganisation, exposure to violence). Participants also responded to items on their personality, offending behaviour, and sexual and mental health. Tables 2–4 outline in detail the waves (respondent age and academic school year) in which participants responded to particular measures. The measures completed by their parents and siblings in the family study are outlined in Tables 5 & 6.

**Table 2:** Socio-demographic, personality and mental health measures by wave.

	Study wave						
	1	2	3	4	5	6	7
Year	2001	2002	2003	2004	2005	2006/2007	2010/2011
Age	11	12	13	14	15	16/17	20/21
School year	8	9	10	11	12		
Socio-demographic							
Gender	√	√	√	√	√	√	√
Marital status						√	√
Family structure	√	√	√	√	√	√	√
Children & pregnancies						√	√
Family SES	√	√	√	√	√		
Disposable income	√	√	√	√	√		
Car ownership						√	√
Employment						√	√
Sources of income						√	√
Weekly hours worked						√	√
Personality							
Impulse control			√				√
Risk taking			√				√
Emotional stability			√				√
Mental health							
SDQ	√			√			
SMFQ					√	√	
PHQ-9							√
PSQ					√	√	√
Self-harming					√	√	√
Use of services					√	√	√
Use of medication					√	√	√

SES = Socio-Economic Status; SDQ = Strengths and Difficulties Questionnaire; SMFQ = Short Mood and Feelings Questionnaire; PHQ-9 = Depression scale of the Patient Health Questionnaire; PSQ = Psychosis Screening Questionnaire

**Table 3:** Family dynamics/processes, substance use, offending & crime and relationship measures by wave.

	Study wave						
	1	2	3	4	5	6	7
Year	2000/2001	2002	2003	2004	2005	2006/2007	2010/2011
Age	10/11	12	13	14	15	16/17	20/21
School year	8	9	10	11	12		
Family dynamics/processes							
IPPA—Parent scale	√		√	√			
Parental monitoring*	√	√	√	√	√		
Arguments with parents					√		
Substance use							
Frequency	√	√	√	√	√	√	√
Location	√	√	√	√	√	√	
Alcohol/drug related problems			√	√			
AUDIT					√	√	√
Drinking motives	√	√	√	√	√	√	√
DAST					√	√	√
CAST							√
Offending & crime							
Offending behaviour	√	√	√	√	√	√	√
Contact with CJS			√	√	√	√	√
Running away from home	√	√	√	√	√		
Relationships							
Sexual history/health						√	√
Substance use by partners							√
ECR-R						√	

IPPA = Inventory of Peer and Parental Attachment;

* = Stattin & Kerr’s Parental Monitoring scale (4 sub-scales- monitoring, solicitation, control & child disclosure);

AUDIT = Alcohol Use Disorders Identification Test; DAST = Drug Abuse Screening; CAST = Cannabis Abuse Screening Test; CJS = Criminal Justice System; ECR-R = The Experiences in Close Relationships—Revised, Questionnaire

**Table 4:** Peer relationships, leisure activities, school & education and neighbourhood characteristics variables by wave.

	Study wave						
	1	2	3	4	5	6	7
Year	2000/2001	2002	2003	2004	2005	2006/2007	2010/2011
Age	10/11	12	13	14	15	16/17	20/21
School year	8	9	10	11	12		
Peer relationships							
Best friend in school year*	√	√	√	√	√		
1–9 other friends in school year*	√	√	√	√	√		
IPPA- Peer scale	√	√					
Relationship with older friends	√	√	√	√	√		
Frequency of friend contact							√
Friends’ substance use (SR)							√
Leisure activities							
Activities (e.g. sport)	√	√	√	√	√		√
Internet use						√	√
Musical tastes	√	√	√				
Household chores					√		
Number of evenings out of the home	√	√	√	√	√		
School & education							
Behaviour in school	√	√	√	√	√		
Attitude to school	√	√	√	√	√		
Educational aspirations	√	√	√	√	√		
Drugs education in school	√	√	√	√	√		
Educational achievement						√	√
Tertiary education							√
Neighbourhood characteristics							
Social control	√	√	√	√	√		
Disorganisation	√	√	√	√	√		
Collective efficacy	√	√	√	√	√		
Neighbourhood attachment	√	√	√	√	√		
Exposure to community violence					√	√	
Neighbourhood social capital							√

* = Social Network data;

IPPA = Inventory of Peer and Parental Attachment; SR = self-reported;

**Table 5:** Socio-demographic, mental health, family dynamics/processes, substance use, offending, rule breaking & crime data collected from (a) parent (s) and/or sibling in the family survey.

Variable description	Family member	
	Parent	Sibling
Socio-demographic		
Gender	√	√
Marital status	√	
Family structure	√	
Number of children	√	
Family SES	√	
Disposable income		√
Car ownership	√	
Employment	√	√
Sources of income	√	√
Mental health		
SDQ	√*	
Impact of child difficulties on family	√	
SMFQ	√*	
Family dynamics/processes		
IPPA- Parent scale		√
Parental monitoring ǂ	√	
Family stress	√	
Family conflict and resolution	√	
Marital satisfaction scale	√	
Substance use		
Frequency	√	√
Alcohol/drug related problems	√	√
AUDIT	√	√
DAST		
Knowledge of BYDS participants’ substance use	√	√
Family sanctions/approval of substance use	√	
Offending, rule breaking & crime		
Offending behaviours	√*	√
Contact with the criminal justice system	**√**	**√**

*Note*. The family survey data were collected during waves 4 & 5 (year 2004–2005) which straddled the academic school years 11 & 12 for the BYDS index child (then aged 14–15 years old).

* = parent report of child’s behaviour;

SDQ = Strengths and Difficulties Questionnaire; SMFQ = Short Mood and Feelings Questionnaire; IPPA = Inventory of Peer and Parental Attachment;

^ǂ^ = Stattin & Kerr’s Parental Monitoring scale (4 sub-scales- monitoring, solicitation, control & child disclosure);

AUDIT = Alcohol Use Disorders Identification Test; DAST = Drug Abuse Screening Test

**Table 6:** Relationship, leisure activity, school & education and neighbourhood characteristic data collected from a parent/s and/or sibling in the family survey.

Variable description	Family member	
	Parent	Sibling
Relationships		
Relationship with BYDS sibling		√
Leisure activities		
Activities (e.g. Sports)		√
School & education		
Attitude to school	√	
Educational aspirations (for child)	√	
Educational achievement	√	
Neighbourhood characteristics		
Social control		√
Disorganisation & efficacy		√
Neighbourhood attachment		√
Community violence		√
Neighbourhood social capital		√

*Note*. The Family survey data were collected during waves 4 & 5 (year 2004–2005) which straddled the academic school years 11 & 12 for the BYDS index child (then aged 14–15 years old).

#### Substance use measures

In each wave (from 10–21 years old), participants reported past month, previous 12 months and lifetime use of a range of substances including alcohol, cigarettes, cocaine, ecstasy and heroin. New items were included over the years to reflect trends in substance use, for example wave 7 (when respondents were aged 20/21 years of age) included items on Mephedrone. Cohort members were screened for alcohol related problems/excessive drinking using the Alcohol Use Disorders Identification Test (AUDIT) [23] a ten item scale which covers the domains of hazardous alcohol use, dependence symptoms and harmful alcohol use. Each item is scored between zero and four, giving a maximum score of 40. Scores in the range of 8–15 represent medium levels of alcohol problems while scores of 16 and above represent high levels of alcohol problems [23]. Participants also completed a four factor, 20 item measure on drinking motives in adolescence [24]. Drug related problems were assessed using the Drug Abuse Screening Test (DAST-20), a 20-item drug abuse screener [25] and the Cannabis Abuse Screening Test (CAST) [26], a 6-item scale which measures cannabis abuse in the general population.

## Results

The BYDS datasets have resulted in a number of publications over the years, the key results to date, along with new findings are summarised in the following thematic sections. A list of all publications are detailed at the BYDS webpages http://www.qub.ac.uk/research-centres/YDS/.

### Sample characteristics

Sociodemographic characteristics are presented in Table 7. Gender was relatively evenly distributed during the ‘school years’ (waves 1–5) while greater proportions of females participated in waves 6–7 (57 and 59 per cent, respectively). Information on free school meals were collected as an indicator of family socio-economic status. On average, one quarter of the sample were in receipt of free schools meals across the first three waves of data collection while approximately one fifth received free school meals in waves 4 and 5.

**Table 7:** Sociodemographic characteristics.

	Study wave						
	1	2	3	4	5	6	7
Year	2001	2002	2003	2004	2005	2006/2007	2010/2011
Age	11	12	13	14	15	16/17	20/21
School year	8	9	10	11	12		
Gender (%)							
Male	55.03	47.59	47.70	47.14	46.96	42.40	40.79
Female	44.91	52.31	52.08	52.51	52.94	57.43	59.21
No response	0.05	0.09	0.22	0.35	0.10	0.17	na
Receipt of free school meals (%)							
	24.67	25.81	23.31	20.03	19.50	na	na
**Base (n)**	**3,834**	**4,343**	**4,522**	**3,965**	**3,830**	**2,335**	**2,087**

Note. Percentages are calculated from Base N (total number of respondents).

The vast majority of the sample attended schools in Belfast; twelve schools participating in the study were located in an intermediate townland with rural catchment areas. Postcode data (home address) provided by participants (in wave 6), indicated 32 per cent of the sample were in the bottom area deprivation quintile, that is, the bottom 20 per cent of the Northern Irish population. At age 11, 37 per cent of participants (n = 1,436) reported there was a lot of crime in their area. By 16/17 years of age, 15 per cent (n = 339) reported a family member had been robbed or attacked in the previous 12 months. Twenty-nine per cent (n = 687) indicated they knew someone (other than a family member) who had been beaten up or attacked in the previous 12 months. Most participants completed their school education at 18 years of age (65 per cent, n = 1,323). A smaller proportion left school at 16 years of age (18 per cent, n = 361). At 20/21 years of age (wave 7), respondents were asked to report on their educational qualifications to date: the majority (71 per cent, n = 1,439) reported they had school leaving qualifications (i.e. A-level qualifications) and fifty-seven per cent (n = 1,154) were either currently studying for or had recently completed a university degree.

### Patterns of substance use

Lifetime use of alcohol, tobacco and cannabis across the seven waves (respondents aged 10–21 years old) are reported in Table 8. In wave 7 (when participants were aged 20/21 years old) lifetime use of substances were as follows: alcohol (94 per cent), tobacco (70 per cent), cannabis (45 per cent). Analyses of BYDS to date highlighted a “hidden” high-risk group of adolescents who although attending school regularly, reported high frequency of cannabis use, which was also associated with use of “hard” drugs (e.g. cocaine) and higher levels of antisocial behaviour [27]. The study also focused on cocaine use: results revealed increasing frequency of reported lifetime use of cocaine in the final years of post-primary education (age 13 to 16): cocaine use was associated with social deprivation and being from disrupted families [28]. Analyses also revealed a positive association between the amount of money young people received and higher rates of drug use [28]. While illegal drug use has, largely, been declining in the UK over the past decade [29], this period has witnessed the emergence of a range of new, mostly synthetic substances that mimic many of the effects of “traditional” drugs. These are known as “legal highs”, or new or novel psychoactive substances (NPS). In the most recent BYDS wave (respondents aged 20/21 years old) we included questions on the then legal high mephedrone and use of other pills. The BYDS data are currently being used to examine categories of drug use (e.g. NPS) and the predictors of drug use class.

**Table 8:** Lifetime substance use by study wave.

	Study Wave						
	1	2	3	4	5	6	7
Year	2000/2001	2002	2003	2004	2005	2006/2007	2010/2011
Age	10/11	12	13	14	15	16/17	20/21
School year	8	9	10	11	12		
Lifetime alcohol use (%)							
	67.9	79.1	86.6	90.8	93.0	91.5	94.0
Lifetime tobacco use (%)							
	37.5	53.1	62.7	67.3	69.4	69.4	70.3
Lifetime cannabis use (%)							
	8.1	20.4	32.8	42.4	46.0	45.0	45.0
**Base (n)**	**3,834**	**4,343**	**4,522**	**3,965**	**3,830**	**2,335**	**2,087**

Note. Percentages are calculated from Base N (total number of respondents).

### Patterns of offending

The data have also been used to investigate offending behaviour among the sample [20]. The results suggest that high offenders are characterised by higher levels of alcohol and cannabis use. Latent Class Growth Analysis results indicated young people were increasingly involved in offending behaviour in the first years of post-primary education, peaking at 13/14 years old with behavioural problems in early adolescence indicating at-risk categories. Most of the adolescent offenders desisted from offending by late adolescence and the key processes that consistently discriminated between persisting and desisting offenders were family dynamics such as parent-child attachment and parental monitoring.

### Family and peers

**Fig 1:**
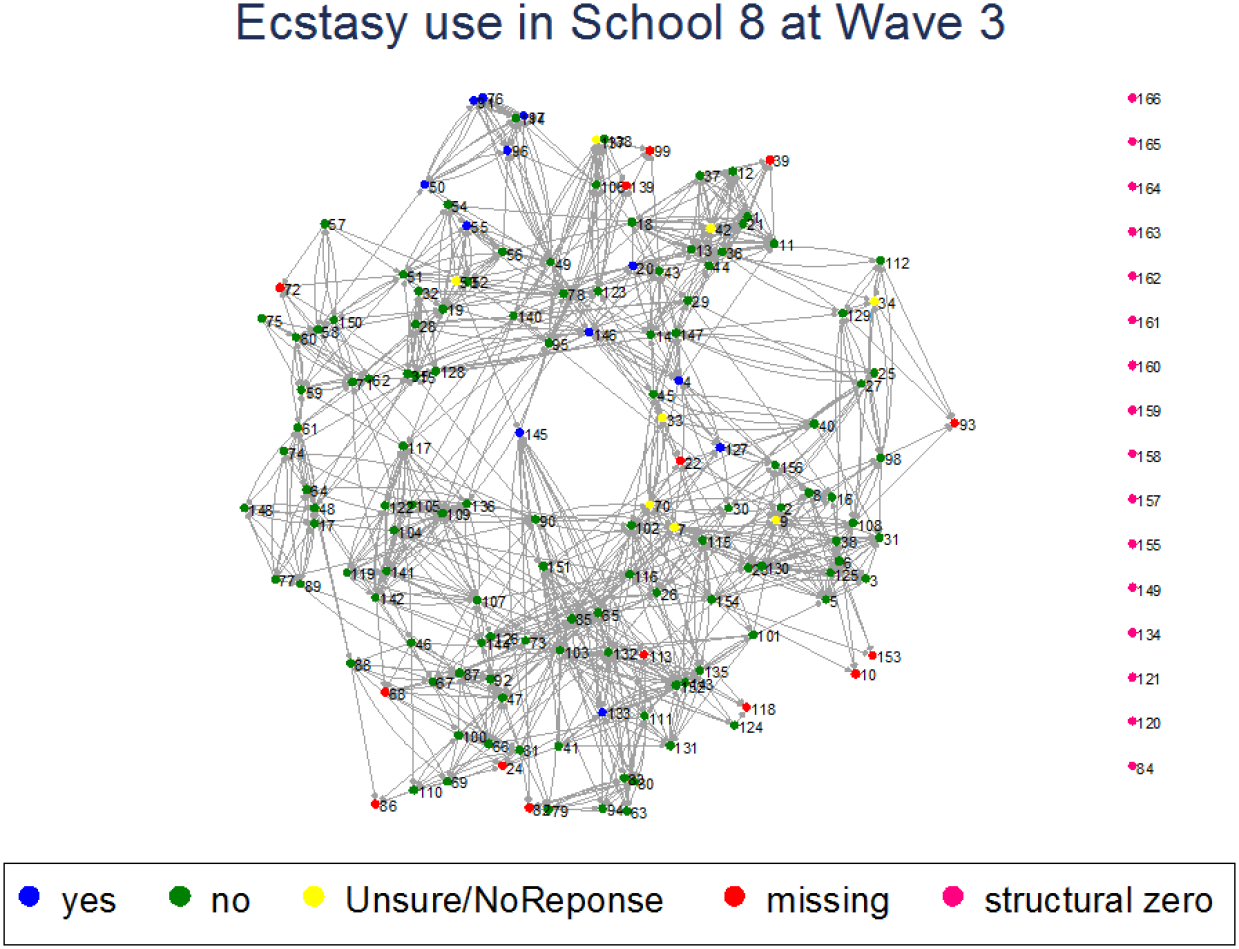
Network model showing spread of ecstasy use in one school in wave 3 (school year 10; respondents aged 13 years old).

**Fig 2:**
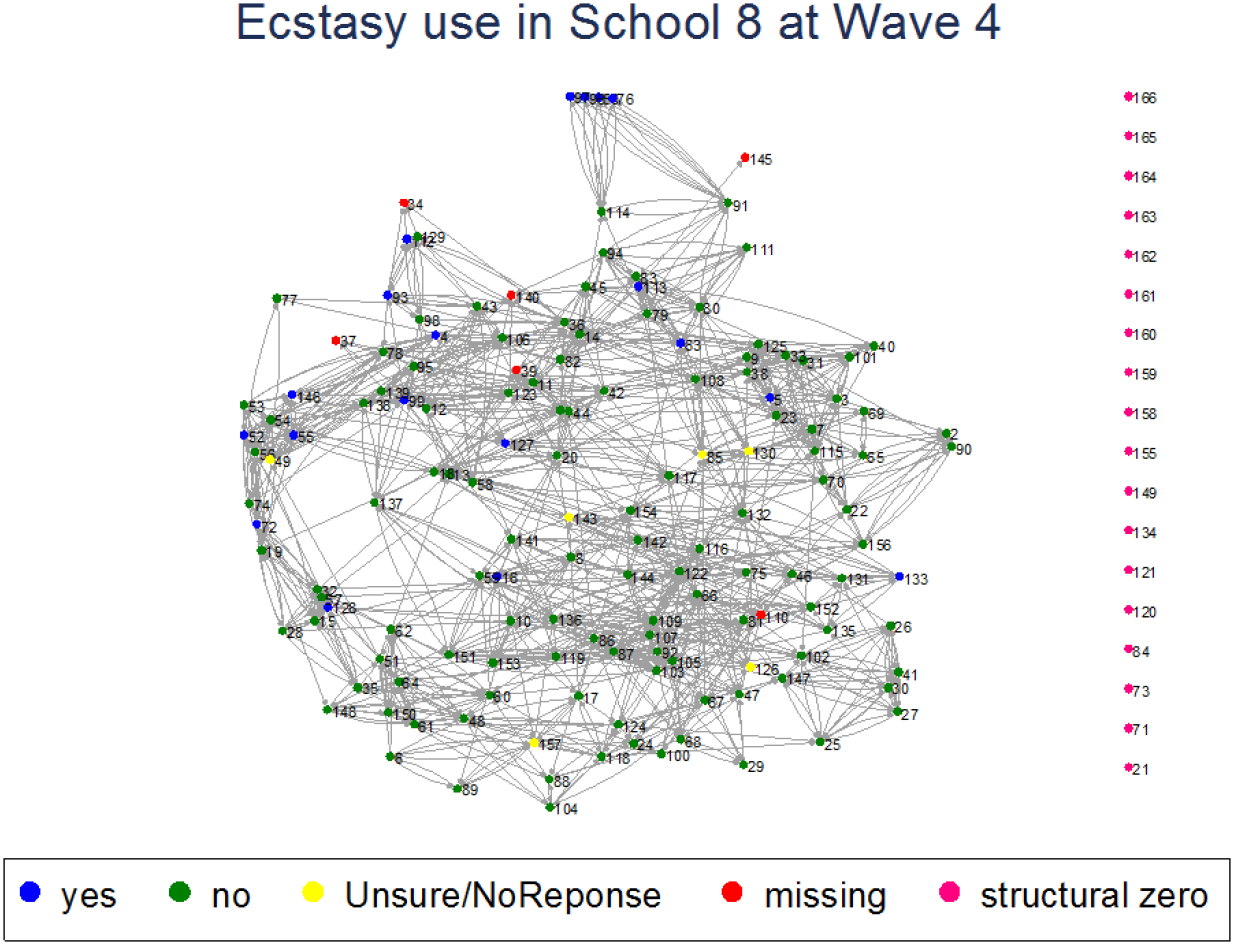
Network models showing spread of ecstasy use in one school in wave 4 (school year 11; respondents aged 14 years old).

The BYDS data have also been used to investigate the complex relationship between family dynamics and adolescent substance use. Analyses investigating elements of a family approach to reducing adolescent drinking frequency indicate greater parental control (i.e. setting and ‘enforcing’ rules) is associated with less frequent adolescent drinking, while parent-child attachment and parental solicitation have little influence [30]. Analyses also indicate exposure to a parent/carer’s drinking when aged 14 is associated with offspring’s subsequent drinking from mid-adolescence, extending into early adulthood [31]. Social network analysis of cannabis use across three waves found that use varied depending on the stability of the friendship network and degree of reciprocity and interconnectedness within the group, concluding that preventing an individual from using cannabis was likely to have a multiplier effect on classmates [32]. Modelling based on the social network data also found evidence that cross-sectional data can be used to estimate peer effects on cannabis use [33]. Other recent findings indicate that while ecstasy use during adolescence may be associated with poorer mental health, the association can be explained by the confounding social influence of family dynamics [34]. The BYDS data have also allowed for the investigation of ecstasy use within a peer/school context. Figs 1 and 2 provide an example of how the network data have been used to plot the spread of ecstasy use in waves 3 and 4 (school years 10–11; respondents aged 13–14 years old) within one particular school.

### Schools

Findings suggest a longitudinal association between school-related variables such as relationships with teachers and a safe school environment and substance use [35]. Analyses conducted on young people excluded from school revealed a higher propensity for drug abuse and antisocial behaviour in this group compared with their peers in mainstream education [27, 36]. Recent analyses [37] identified three distinct profiles of adolescent drinking: late onsetters, steady increasers, minimal users. Regular drinking by mid adolescence had the potential to predict life trajectories by adversely affecting successful transitions between roles and statuses such as gaining employment or commencing training after leaving school.

### Neighbourhoods

Findings suggest associations between exposure to community violence and higher levels of depression, psychotic symptoms, and substance misuse in adolescence [38] and an association between substance use and sexual risk behaviour in late adolescence [39]. BYDS data have also been used to investigate the relationship between affluence, neighbourhood deprivation, drinking patterns and alcohol-related health problems [40]. Adolescent alcohol-related health problems, across patterns of alcohol use profiles, were associated with affluence, whereby being from a deprived background was associated with a reduction in alcohol problem risk for adolescents who demonstrated more hazardous drinking patterns.

## Discussion

The BYDS has collected prospective longitudinal data on a wide range of domains from adolescents during a pivotal developmental period, that is, the transition from secondary school to employment or further education and the transition into “early adulthood”. Findings from analyses to date have provided insight into a range of the key domains interacting with adolescent substance use including family, schools, peers and neighbourhoods. Although BYDS is not a prevalence study, lifetime use of substances among the BYDS cohort are in line with wider UK reports. Lifetime prevalence of cannabis use among 16–24 year olds in the UK has been reported at 40.1 per cent [41], marginally lower than the BYDS cohort (45 per cent) at age 20/21 in 2010/2011. These lifetime reports may indicate the BYDS cohort are no different to international cohorts, despite the post-conflict context.

The peace process in Northern Ireland has led to a normalised Northern Irish society, with a dismantling of the State security apparatus (reduced police numbers and the withdrawal of a military presence on the streets of Northern Ireland), the devolution of certain executive powers to the new Northern Ireland Assembly, the decommissioning of paramilitary weapons (including the disbanding of some paramilitary organisations) and a rapid growth in the Northern Irish night time economy. One of the downsides to the largely positive social changes within Northern Ireland that accompanied this normalisation was the increase in illicit drug consumption and availability to levels similar to those noted across the UK (see [21]).

The BYDS has a number of strengths. The prospective cohort design allows one to follow adolescents before the initiation of problem levels of substance use, and over a long period of time (10 years) enabling the identification of risk and protective factors. Data collection consisted of closely paced waves in schools during the first phase of the study with low rates of refusals and absenteeism during data collection waves. It was particularly valuable to recruit within schools as this provided a simpler way to capture the cohort but also a useful and meaningful unit of analysis (e.g. provision of social network data was facilitated by restricting the scope of this analysis to friends in the same school year who were also sampled in the study). As respondents were clustered within schools and the vast majority of pupils in the school year took part, data were collected which allows for meaningful investigation of contextual effects via multilevel modelling. Respondents nominated their friends within the school year in the first five waves of data collection (school years 8–12; respondents aged 10–15 years old): since these friends were also in the study it is possible to investigate the development of social networks and how these affect and are affected by individual and group characteristics and behaviour. Participants provided self-reported information on a wide range of domains (e.g. mental health; individual characteristics; family processes; behaviour at school and outside school; etc.). The related family study allows for cross-validation of some constructs by investigating multi-informant agreement and further analyses of contextual influences on behaviour. While of international relevance generally, data were also collected during a period of transition to a post-conflict society which may be relevant for other post-conflict/post-trauma groups.

The BYDS is quite distinct from the existing national birth cohort studies within the UK and Ireland (such as the Millennium Cohort Study or the Growing up in Ireland Study). It has a specific focus on the development of adolescent anti-social behaviour (alcohol, drug use and offending behaviours) rather than the broader social and health development of children in the UK and Ireland that is the core of the national cohort studies. It also has a highly geographically clustered sample to facilitate analysis of neighbourhood level influences on adolescent behaviour, and collects data from whole year groups within schools to facilitate the analysis of school level effects and the dynamic social networks that pupils form with their school peers. As a result, it provides a useful addition to the longitudinal data resources available for the study of adolescent development, complementing existing national birth cohort studies and other regional/city based cohort studies (for example The Avon Longitudinal Study of Parents and Children and the Edinburgh Study of Youth Transitions and Crime).

Weaknesses of the BYDS lie in the high attrition rate after the first five waves (school years 8–12; respondents aged 10–15 years old): and the selectiveness of attrition. Despite its limitations, these can be overcome by using data-linkage. Data linkage is now more common place and government datasets are much more accessible to researchers partly due to the development of organisations such as the Administrative Data Research Network (ADRN). We are exploring the potential to link the dataset to administrative data sources, with a view to increasing the richness of BYDS data.

Future plans for BYDS include an 8th wave of data collection as the cohort enter their late twenties, with a view to continue to follow the cohort in adulthood. Future waves of BYDS will obtain consent from participants for linkage so that the BYDS dataset can be used for an even greater range of and more detailed analysis. Through these actions, BYDS will represent an evolving resource for longitudinal substance misuse research incorporating longitudinal data, social network data and self-report data from young people, their parents and peers.

BYDS was originally established to investigate the initiation, persistence and desistance of substance use and as a result the majority of the analyses and outputs to date have focused on substance use. However, the datasets also include a wealth of information (e.g. sexual health, educational attainment), which, whilst used as covariates in many analyses to date can be used as outcome variables in analyses by researchers with interest in these subject areas.

The data are currently held by the Centre for Evidence and Social Innovation (CESI), Queen’s University Belfast and the data is available on request for use by researchers and postgraduate students. Collaborators and postgraduate students are requested to sign a data agreement that sets out responsibilities in handling and using the released data and ensuring schools and individuals cannot be identified. Researchers interested in collaboration should contact the corresponding author, or e-mail BYDS@qub.ac.uk.
